# Effect of immediate initiation of antiretroviral therapy on risk of severe bacterial infections in HIV-positive people with CD4 cell counts of more than 500 cells per μL: secondary outcome results from a randomised controlled trial

**DOI:** 10.1016/S2352-3018(16)30216-8

**Published:** 2017-01-05

**Authors:** Jemma O'Connor, Michael J Vjecha, Andrew N Phillips, Brian Angus, David Cooper, Beatriz Grinsztejn, Gustavo Lopardo, Satyajit Das, Robin Wood, Aimee Wilkin, Hartwig Klinker, Pacharee Kantipong, Karin L Klingman, David Jilich, Elbushra Herieka, Eileen Denning, Ibrahim Abubakar, Fred Gordin, Jens D Lundgren

**Affiliations:** a Research Department of Infection and Population Health, University College London, London, UK; b Institute for Global Health, University College London, London, UK; c Veterans Affairs Medical Center, Washington, DC, USA; d The George Washington University, Washington, DC, USA; e Oxford Centre for Clinical Tropical Medicine, Nuffield Department of Medicine, Oxford University, Oxford, UK; f The Kirby Institute, University of New South Wales, Sydney, NSW, Australia; g STD and AIDS Clinical Research Laboratory, Evandro Chagas Clinical Research Institute (IPEC), Rio de Janeiro, Brazil; h Fundación Centro de Estudios Infectológicos, Buenos Aires, Argentina; i HIV and GU Medicine, Coventry and Warwickshire Partnership Trust, University of Warwick, Coventry, UK; j Desmond Tutu HIV Centre, Institute of Infectious Disease and Molecular Medicine, University of Cape Town, South Africa; k Section on Infectious Diseases, Wake Forest University School of Medicine, Winston-Salem, NC, USA; l University of Würzburg Medical Center, Department of Internal Medicine II, Würzburg, Germany; m Chaingrai Prachanukroh Hospital, Chaingrai, Thailand; n Division of AIDS, National Institute of Allergy and Infectious Diseases, National Institutes of Health, Bethesda, MD, USA; o Department of Infectious and Tropical Diseases, First Faculty of Medicine, Charles University and Na Bulovce Hospital, Prague, Czech Republic; p Royal Bournemouth Hospital, Bournemouth, UK; q Division of Biostatistics, University of Minnesota, Minneapolis, MN, USA; r Department of Infectious Diseases, Rigshospitalet, University of Copenhagen, Copenhagen, Denmark

## Abstract

**Background:**

The effects of antiretroviral therapy on risk of severe bacterial infections in people with high CD4 cell counts have not been well described. In this study, we aimed to quantify the effects of immediate versus deferred ART on the risk of severe bacterial infection in people with high CD4 cell counts in a preplanned analysis of the START trial.

**Methods:**

The START trial was a randomised controlled trial in ART-naive HIV-positive patients with CD4 cell count of more than 500 cells per μL assigned to immediate ART or deferral until their CD4 cell counts were lower than 350 cells per μL. We used Cox proportional hazards regression to model time to severe bacterial infection, which was defined as a composite endpoint of bacterial pneumonia (confirmed by the endpoint review committee), pulmonary or extrapulmonary tuberculosis, or any bacterial infectious disorder of grade 4 severity, that required unscheduled hospital admissions, or caused death. This study is registered with ClinicalTrials.gov, number NCT00867048.

**Findings:**

Patients were recruited from April 15, 2009, to Dec 23, 2013. The data cutoff for follow-up was May 26, 2015. Of 4685 HIV-positive people enrolled, 120 had severe bacterial infections (immediate-initiation group n=34, deferred-initiation group n=86; median 2·8 years of follow-up). Immediate ART was associated with a reduced risk of severe bacterial infection compared with deferred ART (hazard ratio [HR] 0·39, 95% CI 0·26–0·57, p<0·0001). In the immediate-initiation group, average neutrophil count over follow-up was 321 cells per μL higher, and average CD4 cell count 194 cells per μL higher than the deferred-initiation group (p<0·0001). In univariable analysis, higher time-updated CD4 cell count (0·78, 0·71–0·85, p=0·0001) was associated with reduced risk of severe bacterial infection. Time-updated neutrophil count was not associated with severe bacterial infection. After adjustment for time-updated factors in multivariable analysis, particularly the CD4 cell count, the HR for immediate-initiation group moved closer to 1 (HR 0·84, 0·50–1·41, p=0·52). These results were consistent when subgroups of the severe bacterial infection composite were analysed separately.

**Interpretation:**

Immediate ART reduces the risk of several severe bacterial infections in HIV-positive people with high CD4 cell count. This is partly explained by ART-induced increases in CD4 cell count, but not by increases in neutrophil count.

**Funding:**

National Institute of Allergy and Infectious Diseases National Institutes of Health, Agence Nationale de Recherches sur le SIDA et les Hépatites Virales, Bundesministerium für Bildung und Forschung, European AIDS Treatment Network, Australian National Health and Medical Research Council, UK National Institute for Health Research and Medical Research Council, Danish National Research Foundation.

## Introduction

The Strategic Timing of AntiRetroviral Treatment (START) trial, in people with CD4 counts of more than 500 cells per μL and who were naive to antiretroviral therapy (ART), revealed a 57% reduction in risk of AIDS and non-AIDS morbidity and mortality in participants randomly assigned to immediate ART initiation compared with those allocated to deferred initiation.^1^ HIV treatment guidelines were subsequently rewritten to recommend that people living with HIV should be offered ART at any CD4 cell count.^1^, ^2^, ^3^, ^4^ The primary article for the START study reported a reduced risk of bacterial infectious disorder events and incidence of tuberculosis in the immediate-initiation group. Characterisation of the effect of early ART on the incidence of bacterial infections is important because these events are common in people living with HIV.^5^, ^6^, ^7^, ^8^, ^9^, ^10^

In the START study, most serious AIDS-related events, serious non-AIDS-related events, or deaths occurred in patients with CD4 counts above 500 cells per μL and the treatment effect was only partly mediated by changes in CD4 cell count during follow-up.^1^ This finding suggests that ART has beneficial effects on the immune system beyond those measured with CD4 cell count. Neutrophils play a key part in the innate immune response to bacterial infection.^11^ Known as the hallmark of inflammation, neutrophils are the first of the white blood cells to migrate towards a site of bacterial infection to begin killing the invading microbes.^12^ Neutrophils recruit and activate monocytes, dendritic cells, and lymphocytes^13^, ^14^ and are also responsible for confining the pathogen to the local site, thus preventing systemic spread of bacterial disease.^15^ We therefore hypothesised that ART increases the number of neutrophils, which might mediate the association between ART and reduced risk of severe bacterial infection. We also aimed to characterise the factors associated with risk of severe bacterial infection in this multiregional study of people with high CD4 cell counts.

**Research in context**

**Evidence before this study**

We searched the Web of Knowledge for search terms “randomi*” AND “trial” AND “antiretroviral” AND “bacter*” AND “infection*”, AND “HIV*” on April 1, 2016, for articles published in English only. We found no substantive randomised trial evidence published about the effect of antiretroviral therapy (ART) on risk of severe bacterial infections in people with HIV and high CD4 cell count (>500 cells per μL) before this study.

**Added value of this study**

The START trial was a trial of immediate versus deferred ART in 4685 participants with HIV and high CD4 cell count who were followed up for a median of 2·8 years. Our results show that ART has a substantial beneficial effect on bacterial infections, which is largely mediated by improvements in CD4 cell count in people on ART. This benefit was found also for non-tuberculosis bacterial infections. Although there were improvements in neutrophil count in the immediate ART group compared with the deferred ART arm, time-updated neutrophil count did not predict the risk of bacterial infections.

**Implications of all the available evidence**

ART has a protective effect in reducing the risk of a broad spectrum of severe bacterial infections in people with HIV, even in people with high CD4 cell count. This finding further emphasises the benefits of ART even in people with high CD4 cell count.

In this Article, we report the effects of immediate ART on all severe bacterial infections. Our aim was to investigate whether the reduction in severe bacterial infection observed in the immediate-initiation group could be explained by possible increases in neutrophil counts, and the increases in CD4 cell counts that were observed in this group (as reported previously^1^), and to investigate other factors associated with the incidence of severe bacterial infection. The analysis of the effect of treatment group on incidence of severe bacterial infection as a secondary outcome was preplanned, but the choice of the composite definition of severe bacterial infection and the analysis of the extent to which the effect might be mediated by changes in biomarkers was not preplanned.

## Methods

### Study design and participants

The START study was a multicontinental, randomised trial designed to investigate the risks and benefits of immediate initiation of ART in asymptomatic HIV-positive people. Participants with CD4 cell counts above 500 cells per μL were randomly assigned either to start ART immediately or to defer ART until their CD4 cell counts dropped to 350 cells per μL, or until the development of an AIDS-defining event or another condition (eg, pregnancy) that required treatment with ART. Enrolment began on April 15, 2009, and ended on Dec 23, 2013. 4685 HIV-positive people were enrolled (immediate-initiation group, n=2326; deferred-initiation group, n=2359) at 215 clinical sites caring for people living with HIV in 35 countries (the full list of countries has been reported previously^1^). On May 15, 2015, the data safety monitoring board concluded that the primary outcome in START had been achieved.^1^ The data cutoff for follow-up was May 26, 2015.

The study was approved by the institutional review board or ethics committee at each participating site, and written informed consent was obtained from all patients.^1^

### Procedures

People were followed up 1 month and 4 months after randomisation and every 4 months thereafter for data collection and routine follow-up clinical evaluation. Total follow-up time was about 14 060 person-years, with a mean follow-up time of 3·0 years.^1^ Details of the design and data collection methods of the START study have been published elsewhere.^1^, ^16^ CD4 cell count and HIV RNA were measured at randomisation, at 1 month and 4 months after randomisation, and every 4 months thereafter; all other laboratory markers were measured at randomisation and annually thereafter. A severe bacterial infection consisted of any of bacterial pneumonia, pulmonary or extrapulmonary tuberculosis (as reviewed by the INSIGHT endpoint review committee and confirmed or designated as probable), or any bacterial infectious disorder (as reported previously^1^) that met at least one of the three criteria: grade 4 severity, requiring unscheduled hospital admission, or causing death. Follow-up was censored at the time of the severe bacterial infection event, the date of the last study visit, the date of death, or the withdrawal date for participants who withdrew from the study (censoring date for the analysis). The trial protocol has been published previously.^1^

### Statistical analysis

We calculated summary statistics of baseline and time-updated characteristics for all participants. We calculated time-updated summary statistics by taking the mean value of the characteristic for each person over the observed follow-up period (until the censoring date) and summarising the mean of all individuals.

We plotted the change in neutrophil count, total lymphocyte count, haemoglobin concentration, platelet count, albumin concentration, and CD4 cell count over time at annual timepoints, on the basis of values taken at annual visits. We fitted a longitudinal mixed model with PROC MIXED in SAS (version 9.4) to assess the difference in the mean change in laboratory markers from study entry between the treatment groups. We fitted a separate mixed model for each of the laboratory markers with change since study entry as the dependent variable. We adjusted models for the baseline value of the laboratory marker and the visit number. A variance components covariance structure was specified to model the potential correlation between repeated laboratory measures collected at different visits for the same person.

We used Cox proportional hazards regression to model the time to severe bacterial infection. We estimated univariable and multivariable hazard ratios (HRs) with 95% CIs to assess the association between severe bacterial infection and several covariates.

Baseline categorical covariates investigated include treatment group (immediate-initiation, deferred-initiation), sex, mode of HIV acquisition (men who have sex with men; sex with person of the opposite sex; injection drug use, blood products, other, or unknown) and race or ethnicity (Asian, black, white, Latino or Hispanic, other). Time-updated categorical covariates investigated include body-mass index (BMI; <18·5 kg/m^2^, 18·5–24·9 kg/m^2^, 25·0–34·9 kg/m^2^, ≥35 kg/m^2^), and smoking status. Baseline continuous covariates investigated were age and time since HIV diagnosis. Time-updated continuous covariates investigated include CD4 cell count, HIV RNA viral load (log_10_), neutrophil count, total lymphocyte count, haemoglobin concentration, platelet count, and albumin concentration. We stratified all models by a six-category variable describing the geographic region of residence (Africa, Asia, Australia, Europe and Israel, North America, South America and Mexico). We assessed whether the effect of treatment group was consistent across several predefined subgroups that were defined according to characteristics at study entry, by including terms for interactions between treatment and subgroup variables in expanded Cox models. We also assessed the proportional hazards assumption for the treatment effect by fitting the interaction with log time.

We did several additional analyses to separately analyse some components of the severe bacterial infection endpoint where numbers were sufficient. Analyses were done with SAS software (version 9.4). All tests of significance were two-sided and used p<0·05 as the threshold of statistical significance. The START trial was registered with ClinicalTrials.gov, number NCT00867048.

### Role of the funding source

The funder had a role in study design and interpretation of data and the writing of the report through the membership of KLK (Medical Officer HIV Research Branch, DAIDS) in the Writing Committee. The funder had no role in the decision to submit the paper for publication. The corresponding author had full access to all the data in the study and had final responsibility for the decision to submit for publication.

## Results

4685 HIV-positive START participants enrolled between April 15, 2009, and Dec 23, 2013, were included in the analyses. Most participants were male, of white ethnicity, with same-sex mode of HIV acquisition, living in the region of Europe and Israel, and were non-smokers (table 1).

120 people had at least one severe bacterial infection (immediate-initiation group n=34, deferred-initiation group n=86; table 2) during 13 854 person-years at risk (incidence of 0·87 per 100 person-years of follow-up, 95% CI 0·71–1·02). By geographic region, the incidence of severe bacterial infection was 1·1 per 100 person-years (27 in 2429 person-years) in Africa; 0·7 per 100 person-years (seven in 976 person-years) in Asia; 2·8 per 100 person-years (ten in 356 person-years) in Australia; 0·9 per 100 person-years (44 in 5109 person-years) in Europe and Israel; 1·0 per 100 person-years (17 in 1671 person-years) in North America; and 0·5 per 100 person-years (15 in 3313 person-years) in South America. In a univariable Cox model, compared with South America, the hazard ratio for severe bacterial infection was 2·44 (95% CI 1·30–4·60, p=0·006) in Africa, 1·57 (0·64–3·86, p=0·32) in Asia, 1·92 (1·07–3·45, p=0·03) in Europe and Israel, 2·18 (1·08–4·41, p=0·03) in North America, and 6·19 (2·77–13·79, p<0·0001) in Australia. Subsequent Cox models were stratified by geographic region.

**Figure 1:**
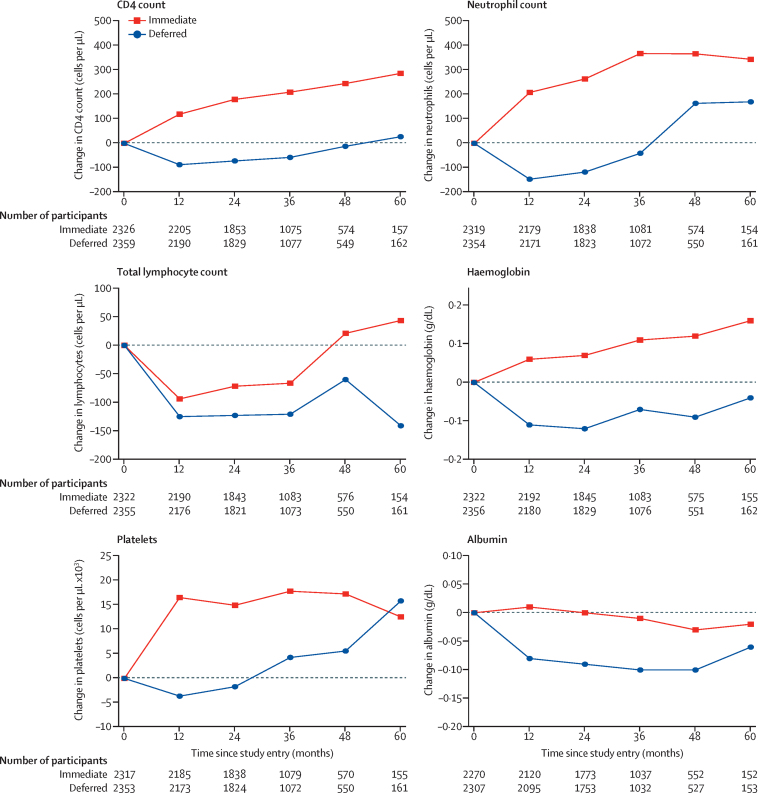
Change in laboratory markers during the study period Longitudinal mixed model adjusted for baseline value of biomarker and visit number. Estimated difference in CD4 count 194·2 cells per μL (95% CI 185·1–203·3; p<0·0001); neutrophil count 321·4 cells per μL (258·6–384·2; p<0·0001); total lymphocyte count 55·3 cells per μL (23·3–87·3; p=0·0007); haemoglobin 0·19 g/dL (0·14–0·24; p<0·0001); platelets 17·8 cells per μL × 10^3^ (15·3–20·2; p<0·0001); albumin 0·09 g/dL (0·07–0·10; p<0·0001).

Baseline median values of biomarkers investigated for their association with severe bacterial infection were: CD4 count 651 cells per μL (IQR 584–764); neutrophil count 2830 cells per μL (2149–3700); total lymphocyte count 2200 cells per μL (1830–2659); haemoglobin concentration 14·5 g/dL (13·4–15·3); platelet count 231 × 10^3^ cells per μL (195 × 10^3^ to 273 × 10^3^); and albumin concentration 4·4 g/dL (4·1–4·6). During follow-up, the change in biomarker values since study entry was significantly higher in the immediate-initiation group than in the deferred-initiation group, for all biomarkers investigated (figure 1).

**Figure 2:**
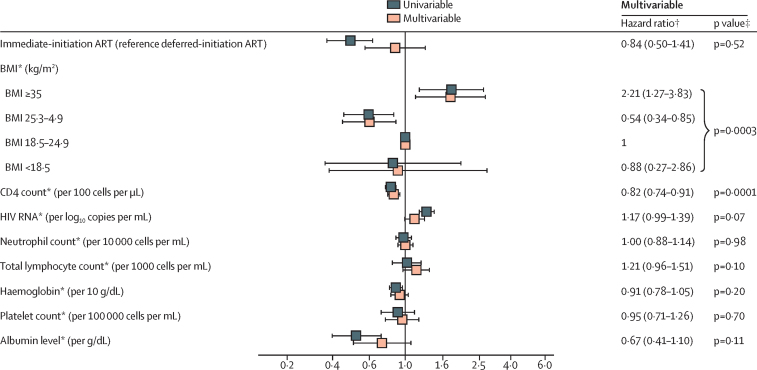
Factors associated with severe bacterial infection Number of events by treatment group for BMI: BMI lower than 18·5 kg/m^2^ (one immediate *vs* two deferred), BMI 18·5–24·9 kg/m^2^ (51 immediate *vs* 17 deferred), BMI 25·0–34·9 kg/m^2^ (20 immediate *vs* 11 deferred), and BMI 25·0 kg/m^2^ or higher (14 immediate *vs* 4 deferred). All models were stratified by a six-category variable describing the geographic region of residence (Africa, Asia, Australia, Europe and Israel, North America, South America and Mexico). Analyses of various subcategories of the composite endpoint are presented in the appendix (pp 2–9) and generated broadly consistent results. *Time-updated covariate. †Multivariable model also includes age (p=0·44), sex (p=0·07; trend towards women having lower risk than men), race or ethnicity (p=0·22), smoking status (p=0·09; trend towards smokers having higher risk than non-smokers), mode of HIV infection (p=0·88), time since diagnosis (p=0·89), hepatitis B (p=0·67), and hepatitis C status (p=0·43). ‡p value from likelihood ratio test.

In univariable analysis, immediate initiation of ART was associated with a reduced risk of severe bacterial infection (HR 0·39, 95% CI 0·26–0·57, p<0·0001; no evidence of lack of proportionality in hazards, p=0·97; figure 2). 43 severe bacterial infection events occurred before ART initiation (41 of which occurred in the deferred-initiation group) in 5195 person-years at risk (incidence of 0·79 per 100 person-years of follow-up) and 77 occurred after ART initiation (45 of which occurred in the deferred-initiation group) in 8659 person-years at risk (incidence of 0·89 per 100 person-years of follow-up).

Of 120 people who developed a severe bacterial infection, 50 had a bacterial infectious disorder (immediate-initiation group n=14, deferred-initiation group n=36), 48 developed bacterial pneumonia (immediate-initiation group n=14, deferred-initiation group n=34), and 26 developed tuberculosis (immediate-initiation group n=6, deferred-initiation group n=20; table 2). These components of severe bacterial infection do not total 120 because some people had more than one condition.

Univariable and multivariable HRs for a selection of factors that were included in the Cox model are presented in figure 2. In multivariable analysis (after adjustment for all covariates listed in the methods including time-updated variables), the HR (immediate-initiation *vs* deferred-initiation) was no longer significantly below 1 (multivariable HR 0·84, 95% CI 0·50–1·41, p=0·52; univariable 0·39; 0·26–0·57, p<0·0001).

In multivariable analysis, a BMI above 35 kg/m^2^ was associated with an increased risk of severe bacterial infection when compared with a reference group of a BMI between 18·5 and 24·9 kg/m^2^ (HR 2·17, 95% CI 1·19–3·95, p<0·0001). Higher time-updated CD4 cell count was associated with a reduced risk of severe bacterial infection (0·78, 0·71–0·85, p=0·0001). The time-updated CD4 cell count remained strongly associated with a reduced risk of severe bacterial infection (0·84, 0·76–0·93, p=0·0006) when time-updated values were delayed by 6 months. Adjustment for time-updated CD4 cell count in the multivariable model was responsible for the attenuation of the HR for treatment group. Time-updated neutrophil count was not associated with risk of severe bacterial infection (HR [multivariable model] 1·00, 95% CI 0·88–1·14, p=0·98); which was also the case when neutrophil count was fitted as a categorical variable. Other factors that were included in the multivariable model, but were not associated with severe bacterial infection, were age (p=0·44), sex (p=0·07; trend towards females having lower risk), race/ethnicity (p=0·22), time-updated smoking status (p=0·09; trend towards smokers having higher risk), mode of HIV infection (p=0·88), time since HIV diagnosis (p=0·89), hepatitis B (p=0·67) and hepatitis C status (p=0·43), time-updated HIV RNA viral load (p=0·07; trend towards higher levels being associated with higher risk), time-updated total lymphocyte count (p=0·10), time-updated haemoglobin concentration (p=0·20), time-updated platelet count (p=0·70), and time-updated albumin concentration (p=0·11). The tests for interaction between the treatment group and several predefined subgroups that were determined by characteristics upon study entry were non-significant.

Given that the beneficial effect of immediate ART on risk of severe bacterial infection seems to be partly mediated by induced rises in CD4 cell count, we considered whether the effect would be lower in people with baseline CD4 cell counts above the median of 651 cells per μL than people with baseline CD4 cell counts below the median, but the univariable effect of immediate ART was similar to the overall effect (HR 0·43; 95% CI 0·24–0·80, p=0·007).

We did four sensitivity analyses to explore how sensitive the results of our study were to variations in the definition of severe bacterial infection. In one analysis, we excluded tuberculosis from the definition of severe bacterial infection (appendix p 2). In a second analysis, additionally, all syphilis events were ignored and follow-up for people who experienced a syphilis event was censored at the last visit date, death, or withdrawal (appendix p 4). Two further sensitivity analyses were done in which bacterial pneumonia and bacterial infectious disorder were treated as separate endpoints. Results of all these analyses are broadly similar to the primary analysis whereby adjustment for time-updated CD4 cell count reduces the HR for treatment group, and high CD4 cell count predicted these endpoints. High BMI was more strongly associated with excess risk of bacterial pneumonia than with bacterial infectious disorders (appendix p 6).

## Discussion

Immediate ART initiation reduced the risk of severe bacterial infections in asymptomatic HIV-positive people with high CD4 cell counts by 61%. The benefit of immediate ART was attenuated in statistical models after adjustment for time-updated CD4 cell count, suggesting that the effect of immediate ART is partly mediated by the increase in CD4 cell count in the immediate ART group and hence this increase in itself or some linked immunological pathway is an important mechanism by which ART reduces risk of severe bacterial infections. By contrast, we found no evidence that the effect was mediated by the increases seen in neutrophil count with early ART, as we initially hypothesised.

Our findings are consistent with those from previous studies, which show the efficacy of early ART in reducing the risk of bacterial diseases.^8^, ^10^ In a randomised controlled trial that compared immediate initiation of ART with deferred ART (according to the most recent WHO criteria, which changed during the course of the study) in HIV-positive people with CD4 cell count greater than 200 cells per μL in Côte d'Ivoire, early ART was associated with a 77% reduced risk of invasive bacterial disease.^8^ Another randomised controlled trial, done in Haiti in 2010, compared early ART (initiation of ART within 2 weeks of enrolment into the study) in people with CD4 counts greater than 200 cells per μL and less than 350 cells per μL, with no history of an AIDS-defining illness. The study used the 2010 WHO criteria for starting treatment, which involved initiation of ART at a CD4 count less than 200 cells per μL or after development of an AIDS-defining event.^10^ Early ART in this study was associated with a 75% reduced risk of death, where ten of 29 deaths that occurred during the study were caused by a bacterial infection. Neither trial reported on analyses describing the extent to which the effect on bacterial infections was mediated by changes in CD4 cell count or neutrophil count.

In accordance with other studies,^17^, ^18^, ^19^, ^20^, ^21^, ^22^ we found an increased risk of severe bacterial infection, particularly for bacterial pneumonia, in people who were obese, and by contrast with other studies,^20^, ^21^ we did not find an increased risk of severe bacterial infection in people who were underweight. In our study, we investigated the effect of adjusting our model for time-updated diabetes to establish whether BMI was acting as a proxy for excess risk of diabetes. We found that diabetes did not attenuate the HR for BMI.

The relationship between BMI and risk of bacterial pneumonia was investigated in a 2013 meta-analysis,^22^ which concluded that the relationship between BMI and pneumonia in the 25 studies included in the meta-analysis was significantly heterogeneous (p<0·0001). Results of the meta-analysis showed a J-shaped relationship between BMI and community-acquired pneumonia, in which people who were underweight had an 80% increased risk of community-acquired pneumonia, being overweight was found to be protective of community-acquired pneumonia, and people who were obese had a 3% increased, but non-significant, risk of community-acquired pneumonia.^22^

Our findings provide further evidence of a clinical benefit of early initiation of ART. Biomarkers in untreated HIV infection tend to decline even in people with high CD4 cell counts (figure 1). Control of viral replication with ART, with consequent benefits on immunological function, at early stages of HIV infection reverses these trends and is important for the prevention of a range of bacterial infections. The benefits of immediate ART were consistent across different subgroups and this beneficial effect of early ART did not differ according to age, sex, race or ethnicity, BMI, smoking status, CD4 cell count, or time since HIV diagnosis. Early ART provides public health benefits by way of a reduced risk of transmission;^23^, ^24^ in this study we show individual-level health benefits in addition to those presented in the START primary publication. Our findings could be of particular importance in regions where a high burden of bacterial diseases might exacerbate the natural history of HIV disease.^25^, ^26^, ^27^, ^28^, ^29^, ^30^, ^31^

The main strengths of our study are the comparison of randomised groups, the use of pre-established standardised methods for data collection, and the size and diversity of the study cohort. All serious events and deaths were reviewed by an endpoint review committee, who were blind to the treatment group, using pre-established INSIGHT criteria.^32^ Endpoints including death from any cause, grade 4 events, and unscheduled hospital admissions for reasons other than AIDS were collected and categorised according to codes used in the Medical Dictionary for Regulatory Activities (version 18.0). Additionally, collation of longitudinal follow-up data from 35 countries with standardised data collection methods provided a large, ethnically, geographically, and socially diverse cohort of HIV-positive people. Furthermore, rich data collection allowed for adjustment of several participant and HIV-related covariates that enabled us to assess potential association of these covariates with severe bacterial infection.

One limitation of this study is that only the bacterial pneumonia and tuberculosis components of our endpoint were reported to and reviewed by the endpoints review committee. Other bacterial infections were identified only if they were classified as grade 4 events and required unscheduled hospital admission or led to death. Extrapolation of our findings to less severe bacterial infections, for which data were absent, should be done with caution. Furthermore, hospital admission might be more likely in some settings than others for a given infection. Indeed, the incidence of severe bacterial infections was notably highest in Australia whereas we might have expected higher rates in Africa, South America, and Asia. Furthermore, our analyses involved few events (n=120), which limits the study power. Additionally, although CD4 cell count and HIV RNA were measured more frequently, all other laboratory markers were measured at randomisation and annually thereafter. Therefore, for severe bacterial infection events that occurred in the first year after randomisation (n=37; eight in immediate ART group and 29 in the deferred ART arm), there would be no follow-up measurement of these laboratory markers. This might have resulted in overestimation of the importance of CD4 cell count in explaining the treatment effect, relative to the other laboratory markers.

In summary, the results of our study show the protective effect of immediate ART in reducing the risk of a broad spectrum of severe bacterial infections in HIV-positive people. The effect of immediate ART appears to be mediated in part by ART-induced increases in CD4 cell count, but not by the ART-induced increases in neutrophil count.

## Figures and Tables

**Table 1:** Patient characteristics

		**Participants who had a severe bacterial infection (n=120)**	**All participants (n=4685)**
Treatment group			
	Immediate-initiation	34 (28%)	2326 (50%)
	Deferred-initiation	86 (72%)	2359 (50%)
Age (years)		38 (29–47)	36 (29–44)
Female sex		34 (28%)	1257 (27%)
Race or ethnicity			
	Black	42 (35%)	1410 (30%)
	White	61 (51%)	2086 (45%)
	Asian, Latino, Hispanic, or other	17 (14%)	1189 (25%)
Geographic region of residence			
	Africa	27 (23%)	1000 (21%)
	Asia	7 (6%)	356 (8%)
	Australia	10 (8%)	109 (2%)
	Europe and Israel	44 (37%)	1539 (33%)
	North America	17 (14%)	507 (11%)
	South America	15 (13%)	1174 (25%)
Route of infection with HIV			
	Men having sex with men	61 (51%)	2586 (55%)
	Sex with person of opposite sex	48 (40%)	1790 (38%)
	Injection drug use, blood products, other, or unknown	11 (9%)	309 (7%)
Time since HIV diagnosis (years)		1·1 (0·4–3·4)	1·0 (0·4–3·1)
Hepatitis B-positive		2 (2%)	128 (3%)
Hepatitis C-positive		9 (8%)	171 (4%)
Time-updated characteristics*†			
	BMI (kg/m^2^)	24·1 (21·8–29·7)	24·7 (22·2–27·9)
	Current smoker	46 (38%)	1334 (28%)
	CD4 cell count (cells per μL)	598 (514–707)	695 (569–837)
	HIV RNA (log_10_ copies per mL)	3·8 (2·6–4·5)	2·4 (1·8–3·7)
	Neutrophil count (cells per μL)	2639 (2061–3700)	2960 (2300–3770)
	Total lymphocyte count (cells per μL)	2200 (1782–2670)	2165 (1841–2560)
	Haemoglobin (g/dL)	14·1 (13·0–15·1)	14·5 (13·4–15·3)
	Platelet count (cells per μL × 10^3^)	240 (196–273)	237 (202–277)
	Albumin (g/dL)	4·3 (4·0–4·5)	4·4 (4·2–4·6)

Data are n (%) or median (IQR). BMI=body-mass index.

* Summary statistics were calculated for numerical time-updated covariates by taking the mean value of the covariate for each person over follow-up and then summarising the mean of all individuals. For those with a severe bacterial infection event, the most recent value before the event is given.

† For covariates presented as time-updated characteristics, the baseline values for all participants (n [%] or median [IQR]) were BMI 24·55 kg/m^3^ (22·1–27·9), current smoker 1496 (32%), CD4 cell count 651 cells per μL (583–764), HIV RNA 4·1 log_10_ copies per mL (2·8–4·6), neutrophil count 2830 cells per μL (2149–3700), total lymphocyte count 2200 cells per μL (1830–2659), haemoglobin 14·5 g/dL (13·4–15·3), platelet count 231 × 10^3^ cells per μL (195–273), albumin 4·4 (4·1–4·6).

**Table 2:** Severe bacterial infection events

	**Immediate-initiation group (n=34)**	**Deferred-initiation group (n=86)**
Arthritis bacterial	0	2
Bacterial pneumonia (reviewed by ERC)	14	34*
Bacterial pyelonephritis	1	0
*Campylobacter* gastroenteritis	1	2
Cellulitis	1	4
Cellulitis staphylococcal	0	1
Endocarditis bacterial	0	1
Erysipelas	2	3
*Escherichia* urinary tract infection	1	1
Furuncle	0	1
Gastroenteritis bacterial	0	1
Gastroenteritis salmonella	1	0
Gastroenteritis *Shigella*	1	2
Meningitis streptococcal	0	1
Pneumococcal bacteraemia	0	1
*Shigella* infection	1	1
Staphylococcal abscess	0	2
Streptococcal bacteraemia	1	0
Streptococcal sepsis	0	1
Syphilis outside CNS	1	7
Syphilis in CNS	1	4
Tonsillitis streptococcal	1	0
Tuberculosis (pulmonary)	6	17
Tuberculosis (extra-pulmonary)	0	3
Typhoid fever	1	1
Total	34	90*

Infections are listed as MedDRA preferred terms. ERC=INSIGHT endpoint review committee.

* Four people had both an ERC review bacterial pneumonia and a bacterial infectious disorder that satisfied one of the following three criteria: grade 4, hospital admission, or death.
