# Supplementary material for: Effect of immediate initiation of antiretroviral therapy on risk of severe bacterial infections in HIV-positive people with CD4 cell counts of more than 500 cells per μL: secondary outcome results from a randomised controlled trial
Source: Lancet HIV. 2017 Jan 5;4(3):e105–12. doi: 10.1016/S2352-3018(16)30216-8 (PMC5337625; doi:10.1016/S2352-3018(16)30216-8)
Supplement: Supplementary appendix [file mmc1.pdf]

THE LANCET
HIV

Supplementary appendix

This appendix formed part of the original submission and has been peer reviewed. We post it as supplied by the authors.

Supplement to: O'Connor J, Vjecha MJ, Phillips AN, for the INSIGHT START study group. Effect of immediate initiation of antiretroviral therapy on risk of severe bacterial infections in HIV-positive people with CD4 cell counts of more than 500 cells per µL: secondary outcome results from a randomised controlled trial. *Lancet HIV* 2017; published online Jan 4. http://dx.doi.org/10.1016/S2352-3018(16)30216-8.

Supplementary Appendix to Manuscript Entitled

"Effect of immediate ART initiation on risk of severe bacterial infections in HIV-positive people with CD4 count > 500 cells/mm^3"

This supplement presents four sensitivity analyses conducted to explore how sensitive the results of our study were to variations in the definition of severe bacterial infection. In Table S1 we exclude tuberculosis from the definiton of SBI. Table S2 presents the results of an analysis in which, in addition, all syphilis events were ignored and follow-up for people who experienced a syphilis event was censored at the last visit date or death. Two further sensitivity analyses were conducted in which bacterial pneumonia and bacterial infectious disorder were treated as separate endpoints. Results of all these analyses are broadly similar to the primary analysis whereby adjustment for time-updated CD4 count attenuates the hazard ratio for treatment group.

Table S1. Factors associated with severe bacterial infection other than tuberculosis (n=94) - Cox proportional hazards regression

	Model (i)[a]		Model (ii)[b]	
**Covariate**	**Hazard Ratio (95% CI)**	**p-value[c]**	**Hazard Ratio (95% CI)**	**p-value[c]**
**Treatment group (ref deferred ART)**				
Immediate ART	0.41 (0.27 - 0.65)	<.0001	0.74 (0.42 - 1.31)	0.30
**Age (per 10 years)**	1.25 (1.03 - 1.52)	0.026	1.17 (0.94 - 1.46)	0.17
**Female sex (ref male)**	1.66 (0.98 - 2.83)	0.071	1.65 (0.71 - 3.81)	0.24
**Race/ethnicity (ref white)**				
Asian	1.62 (0.40 - 6.59)	0.096	2.26 (0.54 - 9.44)	0.14
Black	1.36 (0.71 - 2.60)		1.20 (0.57 - 2.51)	
Latino/Hispanic	0.29 (0.09 - 1.01)		0.31 (0.09 - 1.06)	
Other	1.26 (0.38 - 4.15)		1.35 (0.40 - 4.54)	
**Body mass index[d] (kg/m^2) (ref 18.5 ≤ BMI < 25)**				
BMI < 18.5	0.91 (0.22 - 3.77)	0.0004	0.97 (0.23 - 4.15)	0.001
25 ≤ BMI < 35	0.82 (0.51 - 1.30)		0.80 (0.49 - 1.30)	
BMI ≥ 35	3.59 (1.95 - 6.60)		3.35 (1.72 - 6.50)	
**Smoking status[d] (ref non-smoker)**	1.13 (0.73 - 1.73)	0.59	1.26 (0.79 – 2.00)	0.34
**Mode of Infection with HIV (ref MSM)**				
Sex with person of opposite sex	1.33 (0.79 - 2.25)	0.54	0.65 (0.30 - 1.41)	0.48
Blood products/IDU/other/unknown	1.25 (0.59 - 2.65)		0.69 (0.29 - 1.66)	
**Time since HIV diagnosis at baseline (years)**	1.03 (0.97 - 1.08)	0.33	1.01 (0.95 - 1.07)	0.82
**Hepatitis B status (ref negative)**				
Positive	1.04 (0.25 - 4.26)	0.99	1.08 (0.26 - 4.51)	0.93
Unknown	1.05 (0.38 - 2.90)		0.80 (0.24 - 2.66)	
**Hepatitis C status (ref negative)**				
Positive	1.92 (0.92 - 3.99)	0.28	1.71 (0.77 - 3.82)	0.43
Unknown	1.14 (0.36 - 3.62)		1.39 (0.35 - 5.51)	
**CD4 count[d] (per 100 cells/mm^3)**	0.79 (0.72 - 0.87)	<.0001	0.83 (0.74 - 0.92)	0.0003
**HIV RNA[d] ($\log_{10}$ copies/mL)**	1.33 (1.15 - 1.53)	0.0001	1.07 (0.89 - 1.30)	0.46
**Neutrophil count[d] (per 1000 cells/mm^3)**	0.96 (0.83 - 1.12)	0.60	1.00 (0.85 - 1.16)	0.94
**Total Lymphocyte count[d] (per 1,000 cells/mm^3)**	1.09 (0.85 - 1.39)	0.54	1.25 (1.00 - 1.56)	0.090
**Haemoglobin[d] (per g/dL)**	0.87 (0.76 - 0.99)	0.038	0.96 (0.80 - 1.15)	0.66
**Platelet count[d] (per 100,000 cells/mm^3)**	0.82 (0.58 - 1.16)	0.26	0.90 (0.63 - 1.28)	0.55

Covariate	Model (i)[a] Hazard Ratio (95% CI)	p-value[c]	Model (ii)[b] Hazard Ratio (95% CI)	p-value[c]
**Albumin[d] (g/dL)**	0.51 (0.31 - 0.83)	0.37	0.79 (0.45 - 1.41)	0.10

MSM: men-who-have-sex-with-men; IDU: Injection-drug use.

[a]Model (i) univariable effects of covariates; stratified by geographic region of residence[e].

[b]Model (ii) multivariable effects of covariates; stratified by geographic region of residence[e].

[c]By the Likelihood ratio test.

[d]Time-updated covariate.

[e]Geographic region of residence: Africa, Asia, Oceania, Europe & Israel, North America, South America.

Table S2. Factors associated with severe bacterial infection - ignoring syphilis (n=81) - Cox proportional hazards regression

	Model (i)[a]		Model (ii)[b]	
**Covariate**	**Hazard Ratio (95% CI)**	**p-value**	**Hazard Ratio (95% CI)**	**p-value**
**Treatment group (ref deferred ART)**				
Immediate ART	0.47 (0.29 - 0.75)	0.002	0.87 (0.47 - 1.61)	0.66
**Age (per 10 years)**	1.27 (1.03 - 1.56)	0.02	1.16 (0.92 - 1.47)	0.21
**Female sex (ref male)**	2.02 (1.16 - 3.52)	0.012	1.73 (0.74 - 4.06)	0.21
**Race/ethnicity (ref white)**				
Asian	0.99 (0.14 - 6.78)	0.044	1.39 (0.19 - 9.99)	0.10
Black	1.65 (0.84 - 3.22)		1.34 (0.64 - 3.04)	
Latino/Hispanic	0.22 (0.05 - 0.98)		0.22 (0.05 - 1.00)	
Other	0.95 (0.22 - 4.05)		0.98 (0.23 - 4.24)	
**Body mass index[c] (kg/m^2) (ref 18.5 ≤ BMI < 25)**				
BMI < 18.5	0.57 (0.08 - 4.21)	0.0001	0.59 (0.08 - 4.44)	0.0006
25 ≤ BMI < 35	1.05 (0.64 - 1.72)		1.00 (0.59 - 1.69)	
BMI ≥ 35	4.67 (2.47 - 8.83)		4.24 (2.11 - 8.51)	
**Smoking status[c] (ref non-smoker)**	1.20 (0.76 - 1.91)	0.43	1.39 (0.84 - 2.29)	0.21
**Mode of Infection with HIV (ref MSM)**				
Sex with person of opposite sex	1.72 (0.99 - 2.97)	0.12	0.81 (0.37 - 1.77)	0.27
Blood products, other or unknown	1.68 (0.78 - 3.60)		0.89 (0.37 – 2.18)	
**Time since HIV diagnosis at baseline (years)**	1.02 (0.96 - 1.08)	0.61	0.99 (0.93 - 1.05)	0.68
**Hepatitis B status (ref negative)**				
Positive	1.16 (0.28 - 4.80)	0.88	1.22 (0.29 - 5.13)	0.97
Unknown	1.28 (0.46 - 3.58)		0.97 (0.28 - 3.34)	
**Hepatitis C status (ref negative)**				
Positive	2.32 (1.11 - 4.86)	0.12	2.02 (0.90 - 4.55)	0.31
Unknown	1.35 (0.42 - 4.33)		1.50 (0.37 - 6.16)	
**Latest CD4 count[c] (per 100 cells/mm^3)**	0.82 (0.74 - 0.90)	<.0001	0.85 (0.75 - 0.95)	0.004
**Latest HIV RNA[c] ($\log_{10}$ copies/mL)**	1.31 (1.13 - 1.53)	0.0005	1.11 (0.90 - 1.37)	0.35
**Neutrophil count[c] (per 1000 cells/mm^3)**	0.97 (0.82 - 1.13)	0.67	0.99 (0.84 - 1.17)	0.91
**Total Lymphocyte count[c] (per 1,000 cells/mm^3)**	1.07 (0.82 - 1.41)	0.62	1.21 (0.93 - 1.56)	0.15
**Hemoglobin[c] (per g/dL)**	0.86 (0.75 - 0.99)	0.037	0.98 (0.80 – 1.19)	0.82
**Platelet count[c] (per 100,000 cells/mm^3)**	0.84 (0.58 - 1.21)	0.34	0.87 (0.59 - 1.28)	0.48

Covariate	Model (i)[a] Hazard Ratio (95% CI)	p-value	Model (ii)[b] Hazard Ratio (95% CI)	p-value
**Albumin[c] (g/dL)**	0.54 (0.32 - 0.92)	0.023	0.90 (0.49 - 1.68)	0.75

MSM: men-who-have-sex-with-men; IDU: Injection-drug use.

[a]Model (i) univariable effects of covariates; stratified by geographic region of residence[e].

[b]Model (ii) multivariable effects of covariates; stratified by geographic region of residence[e].

[c]Time-updated covariate.

[d]Geographic region of residence: Africa, Asia, Oceania, Europe & Israel, North America, South America.

Table S3. Factors associated with bacterial pneumonia (n=48) - Cox proportional hazards regression

Covariate	Model (i)[a] Hazard Ratio (95% CI)	p-value	Model (ii)[b] Hazard Ratio (95% CI)	p-value
**Treatment group (ref deferred ART)**				
Immediate ART	0.41 (0.22 - 0.77)	0.006	0.80 (0.36 - 1.77)	0.58
**Age (per 10 years)**	1.16 (0.88 - 1.52)	0.29	1.04 (0.77 - 1.42)	0.79
**Female sex (ref male)**	1.99 (0.99 - 4.01)	0.054	1.26 (0.43 - 3.67)	0.68
**Race/ethnicity (ref white)**				
Asian	1.76 (0.24 - 12.95)	0.33	2.62 (0.34 - 20.36)	0.45
Black	1.50 (0.65 - 3.45)		1.20 (0.45 - 3.19)	
Latino/Hispanic	0.37 (0.08 - 1.71)		0.38 (0.08 - 1.79)	
Other	1.73 (0.39 - 7.75)		1.59 (0.34 - 7.40)	
**Body mass index[c] (kg/m^2) (ref 18.5 ≤ BMI < 25)**				
BMI < 18.5	1.21 (0.16 - 9.25)	<.0001	1.27 (0.16 - 10.11)	0.0004
25 ≤ BMI < 35	1.24 (0.63 - 2.44)		1.40 (0.68 - 2.90)	
BMI ≥ 35	6.92 (3.16 - 15.15)		7.06 (2.92 - 17.10)	
**Smoking status[c] (ref non-smoker)**	1.34 (0.74 - 2.43)	0.34	1.44 (0.75 - 2.77)	0.28
**Mode of Infection with HIV (ref MSM)**				
Sex with person of opposite sex	1.95 (0.97 - 3.89)	0.15	0.84 (0.31 - 2.32)	0.76
Blood products/IDU/other/unknown	1.77 (0.66 - 4.72)		1.05 (0.36 – 3.07)	
**Time since HIV diagnosis at baseline (years)**	0.99 (0.90 - 1.08)	0.74	0.96 (0.88 - 1.05)	0.40
**Hepatitis B status (ref negative)**				
Positive	1.06 (0.14 - 7.79)	0.79	1.01 (0.13 - 7.78)	0.73
Unknown	0.53 (0.07 - 3.96)		0.42 (0.04 - 4.44)	
**Hepatitis C status (ref negative)**				
Positive	2.91 (1.22 - 6.97)	0.10	2.81 (1.06 - 7.45)	0.16
Unknown	0.82 (0.11 - 5.98)		1.53 (0.15 - 16.06)	
**CD4 count[c] (per 100 cells/mm^3)**	0.82 (0.72 - 0.93)	0.002	0.87 (0.75 - 1.01)	0.059
**HIV RNA[c] (log_{10} copies/mL)**	1.37 (1.12 - 1.67)	0.002	1.18 (0.90 - 1.54)	0.24
**Neutrophil count[c] (per 1000 cells/mm^3)**	1.02 (0.85 - 1.24)	0.81	1.01 (0.81 - 1.24)	0.95
**Total Lymphocyte count[c] (per 1,000 cells/mm^3)**	1.01 (0.69 - 1.48)	0.95	1.09 (0.72 - 1.63)	0.69
**Haemoglobin[c] (per g/dL)**	0.86 (0.72 - 1.04)	0.11	0.96 (0.75 – 1.24)	0.77
**Platelet count[c] (per 100,000 cells/mm^3)**	1.11 (0.72 – 1.71)	0.63	1.05 (0.65 – 1.69)	0.84

Covariate	Model (i)[a] Hazard Ratio (95% CI)	p-value	Model (ii)[b] Hazard Ratio (95% CI)	p-value
**Albumin[c] (g/dL)**	0.49 (0.26 – 0.92)	0.026	0.79 (0.36 – 1.76)	0.57

MSM: men-who-have-sex-with-men; IDU: Injection-drug use.

[a]Model (i) univariable effects of covariates; stratified by geographic region of residence[e].

[b]Model (ii) multivariable effects of covariates; stratified by geographic region of residence[e].

[c]Time-updated covariate.

[d]Geographic region of residence: Africa, Asia, Oceania, Europe & Israel, North America, South America.

Table S4. Factors associated with bacterial infectious disorder (n=50) - Cox proportional hazards regression

Covariate	Model (i)[a] Hazard Ratio (95% CI)	Model (i)[a] p-value	Model (ii)[b] Hazard Ratio (95% CI)	Model (ii)[b] p-value
**Treatment group (ref deferred ART)**				
Immediate ART	0.38 (0.20 - 0.70)	0.002	0.71 (0.32 - 1.58)	0.40
**Age (per 10 years)**	1.37 (1.05 - 1.78)	0.020	1.35 (1.00 - 1.83)	0.054
**Female sex (ref male)**	1.39 (0.63 - 3.05)	0.41	2.70 (0.73 – 10.05)	0.14
**Race/ethnicity (ref white)**				
Asian	1.27 (0.18 - 8.98)	0.35	1.59 (0.22 - 11.59)	0.36
Black	0.92 (0.33 - 2.55)		0.89 (0.29 - 2.75)	
Latino/Hispanic	0.19 (0.02 - 1.54)		0.21 (0.03 - 1.72)	
Other	1.55 (0.36 - 6.69)		1.97 (0.45 - 8.71)	
**Body mass index[c] (kg/m^2) (ref 18.5 ≤ BMI < 25)**				
BMI < 18.5	0.73 (0.10 - 5.40)	0.33	0.97 (0.13 - 7.32)	0.26
25 ≤ BMI < 35	0.62 (0.33 - 1.16)		0.57 (0.29 - 1.10)	
BMI ≥ 35	1.50 (0.51 - 4.46)		1.39 (0.44 - 4.37)	
**Smoking status[c] (ref non-smoker)**	0.91 (0.50 - 1.66)	0.76	1.06 (0.56 - 1.99)	0.87
**Mode of Infection with HIV (ref MSM)**				
Opposite sex/blood products/IDU/other/unknown*	0.83 (0.41 - 1.70)	0.62	0.42 (0.14 - 1.27)	0.092
**Time since HIV diagnosis at baseline (years)**	1.06 (0.99 - 1.13)	0.11	1.04 (0.97 - 1.13)	0.28
**Hepatitis B status (ref negative)**				
Positive	0.98 (0.13 - 7.17)	0.87	1.10 (0.15 - 8.21)	0.99
Unknown	1.39 (0.43 - 4.57)		1.07 (0.26 - 4.36)	
**Hepatitis C status (ref negative)**				
Positive	0.83 (0.20 - 3.45)	0.91	0.71 (0.16 - 3.18)	0.85
Unknown	1.29 (0.31 - 5.35)		1.27 (0.23 - 6.97)	
**Latest CD4 count[c] (per 100 cells/mm^3)**	0.74 (0.64 - 0.86)	<.0001	0.77 (0.66 - 0.91)	0.002
**Latest HIV RNA[c] ($\log_{10}$ copies/mL)**	1.35 (1.11 - 1.64)	0.0023	1.02 (0.79 - 1.31)	0.90
**Latest Neutrophil count[c] (per 1000 cells/mm^3)**	0.86 (0.68 - 1.08)	0.20	0.94 (0.76 – 1.18)	0.61
**Latest Total Lymphocyte count[c] (per 1,000 cells/mm^3)**	1.11 (0.79 - 1.54)	0.55	1.37 (1.03 - 1.83)	0.030
**Latest Haemoglobin[c] (per g/dL)**	0.87 (0.73 - 1.05)	0.14	0.98 (0.76 - 1.26)	0.87
**Latest Platelet count[c] (per 100,000 cells/mm^3)**	0.54 (0.32 - 0.90)	0.018	0.70 (0.41 - 1.19)	0.18
**Latest Albumin[c] (g/dL)**	0.55 (0.27 - 1.13)	0.10	0.85 (0.38 - 1.90)	0.70

MSM: men-who-have-sex-with-men; IDU: Injection-drug use.

*Mode of Infection with HIV was re-parameterised for this analysis due to a zero cell which prevented the model from fitting.

[a]Model (i) univariable effects of covariates; stratified by geographic region of residence[e].

[b]Model (ii) multivariable effects of covariates; stratified by geographic region of residence[e].

[c]Time-updated covariate.

[d]Geographic region of residence: Africa, Asia, Oceania, Europe & Israel, North America, South America.

Figure S1. Kaplan Meier plot of cumulative proportion without having experienced SBI by treatment arm.

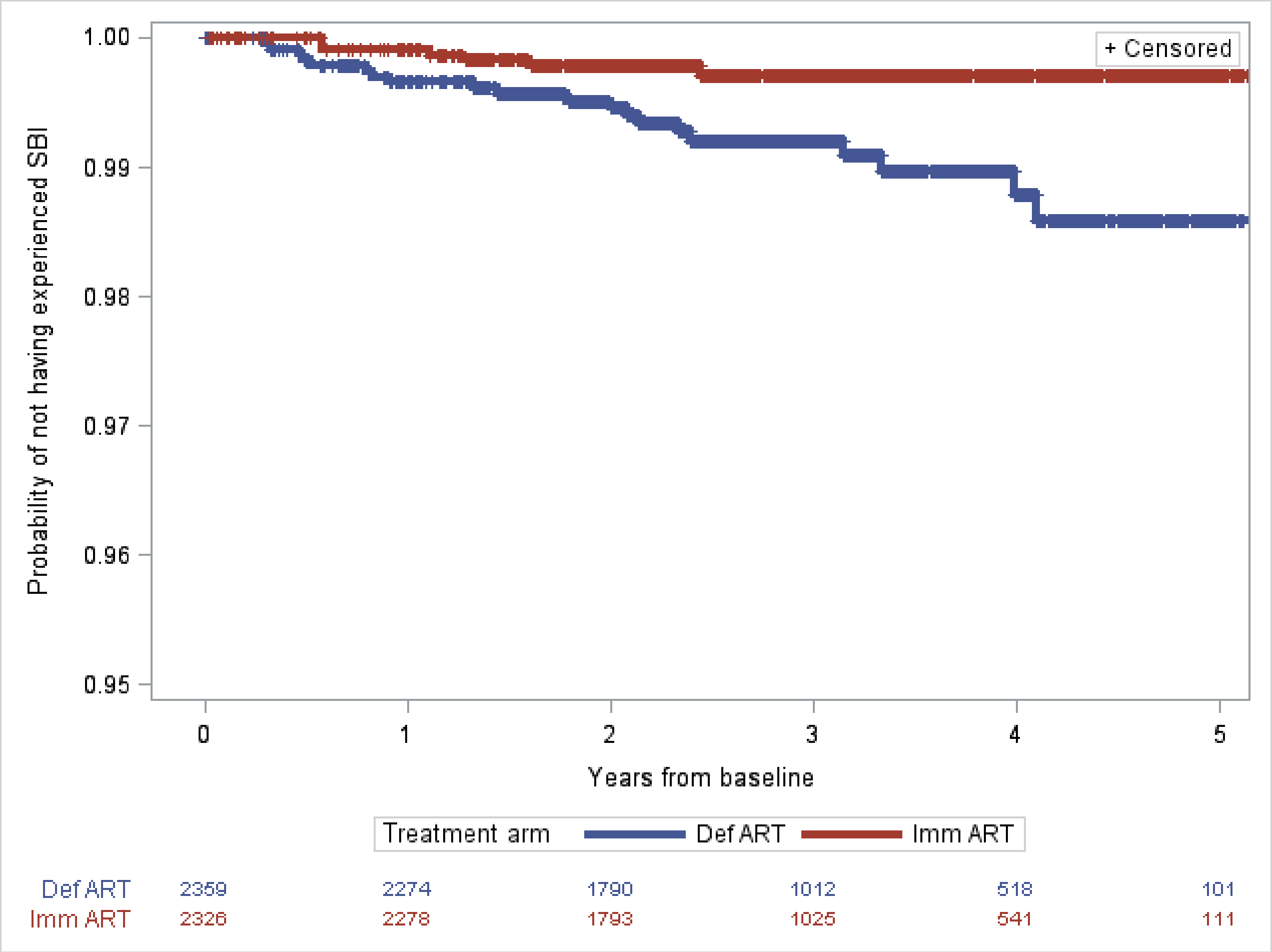

* Timing of SBIs in the deferred arm relative to the start of ART, in the 64 people with an SBI who started ART, were -3 years to - 2 years: 4 (6.3%), -2 years to - 1 years: 8 (12.5%), -1 year to 0 years: 29 (45.3%), 0 years to - +1 years: 12 (18.8%), +1 years to -+2 years: 4 (6.3%), +2 years to +3 years: 5 (7.8%), > +3 years: 2 (3.1%),
